# Normal and malignant epithelial cells with stem-like properties have an extended G2 cell cycle phase that is associated with apoptotic resistance

**DOI:** 10.1186/1471-2407-10-166

**Published:** 2010-04-28

**Authors:** Lisa J Harper, Daniela Elena Costea, Luke Gammon, Bilal Fazil, Adrian Biddle, Ian C Mackenzie

**Affiliations:** 1 Blizard Institute of Cell and Molecular Science, Barts and The London School of Medicine and Dentistry, 4 Newark Street, Whitechapel, London E1 2AT, UK; 2 Section for Pathology, The Gade Institute, University of Bergen, Haukeland University Hospital, N-5021, Bergen, Norway

## Abstract

**Background:**

Subsets of cells with stem-like properties have been previously isolated from human epithelial cancers and their resistance to apoptosis-inducing stimuli has been related to carcinoma recurrence and treatment failure. The aim of this study was to investigate the mechanisms of resistance to apoptosis-inducing agents of cells with stem-like properties in both normal and malignant human epithelia.

**Methods:**

Cells isolated from fresh human head and neck carcinomas (n = 11), cell lines derived from head and neck, prostate and breast human carcinomas (n = 7), and from normal human oral mucosa (n = 5), were exposed to various apoptosis-inducing stimuli (UV, Tumour Necrosis Factor, Cisplatin, Etoposide, and Neocarzinostatin). Flow cytometry for CD44 and epithelial-specific antigen (ESA) expression, colony morphology, tumour sphere formation and rapid adherence assays were used to identify the subset of cells with stem-like properties. Apoptosis, cell cycle and expression of various cell cycle checkpoint proteins were assessed (Western Blot, qPCR). The role of G2-checkpoint regulators Chk1 and Chk2 was investigated by use of debromohymenialdisine (DBH) and siRNA.

**Results:**

In both cancer biopsies and carcinoma cell lines a subset of CD44^high ^cells showed increased clonogenicity, a significantly lower rate of apoptosis, and a significantly higher proportion of cells in the G2-phase of the cell cycle. An inverse correlation between the percentage of cells in G2-phase and the rate of apoptosis was found. Pulse-chase with iododeoxyuridine (IdU) demonstrated that CD44^high ^carcinoma cells spent longer time in G2, even in un-treated controls. These cells expressed higher levels of G2 checkpoint proteins, and their release from G2 with BDH or Chk1 siRNA increased their rate of apoptosis. Low passage cultures of normal keratinocytes were also found to contain a subset of CD44^high ^cells showing increased clonogenicity, and a similar pattern of G2-block associated with apoptotic resistance.

**Conclusions:**

These data indicate that both normal and malignant human epithelial cells with stem-like properties show greater resistance to apoptosis associated with extended G2 cell cycle phase, and that this property is not a consequence of neoplastic transformation. Targeting G2 checkpoint proteins releases these cells from the G2-block and makes them more prone to apoptosis, implying an opportunity for improved therapeutic approaches.

## Background

About one in five US and European deaths is caused by cancer and about four out of five cancer deaths result from cancers of epithelial origin [1-3]. Head and neck squamous cell carcinoma (HNSCC) is the sixth most common malignancy worldwide [4] and, as for other cancers, it is commonly associated with death from tumour recurrence following initial therapy [5]. There is growing awareness that such therapeutic failure may, among other factors, be related to patterns of cellular heterogeneity within tumours [6,7], and the idea that the growth of cancers is associated with a sub-population of cells with stem-like properties, the so called "cancer stem cells" has been discussed for over a century [8]. The continuing growth of malignancies points to the presence of at least some cells with extended self-renewal potential and the usual tumour mimicry of the tissue of origin indicates attempted differentiation of some malignant cells [9]. Thus some tumour cells have the ability for indefinite self-renewal while generating cells that enter differentiation pathways, properties that correspond to the essential basic properties of normal adult somatic stem cells [10]. Further support for this idea has lately been generated by the ability to isolate and assess the tumour-initiating properties of various cell fractions isolated by fluorescence-activated cell sorting (FACS) based on certain cell surface markers such as CD34, CD44 or CD133 [7]. Following the early identification of cells with stem-like properties in haematopoietic malignancies [11,12], prospective identification and isolation of such cell subpopulations has been achieved for an expanding range of solid human tumours, including head and neck, breast and prostate cancers [13-18].

Presence of subpopulations of cells with stem-like properties has also been demonstrated in cell lines derived from various cancers [19-23]. Such cells could be identified *in vitro *not only by high cell surface expression of various markers such as CD44 [20-22], but also by additional, robust methods such as rapid adherence to culture dishes [19] or colony morphology (holoclones, containing small tightly-packed cells vs. meroclones or paraclones, irregular colonies containing large cells) [21,23]. It has recently been shown that their increased *in vitro *clonogenicity correlated well with *in vivo *tumour initiating abilities [22,23].

The primary therapeutic importance of cancer cells with stem-like properties relates to their abilities to resist therapeutic killing in response to chemo- and radio-therapies [7,12,24,25]. Differences in apoptotic sensitivity between the cells with stem-like properties and the rest of the tumour cell population might have therapeutic consequences, the death of mainly the non-stem-like fraction possibly explaining the frequently observed clinical response of early loss of tumour mass followed by later recurrence [10,24,26]. However, although the survival of cells with stem-like properties in some carcinomas has been attributed to an enhanced ability for drug removal, reduced DNA damage, or enhanced DNA repair [24,27,28], the mechanisms behind their differential resistance to apoptosis are not yet clear, nor are they investigated in a broad range of carcinomas or in normal human epithelium. There is a need for more information about the general applicability of such phenomena to carcinoma recurrence, and especially of HNSCC that is characterised by particularly high recurrence rates [29]. Investigating cell populations derived from a quite broad range of carcinomas (head and neck, breast and prostate), and from both fresh tumours and cell lines, the present study shows that the low apoptotic rates consistently observed in the subset of carcinoma cells with stem-like properties are associated with unique cell cycle features that can be therapeutically targeted. Of note is the observation that this seems to be a constitutive trait of cells with stem-cell properties in human adult epithelium and not acquired during progression to malignancy.

## Methods

### Cell isolation from fresh biopsies of normal and malignant human oral mucosa

Tissue was collected from biopsies of normal human oral mucosa (n = 5) and HNSCC tumours (n = 11) with written informed patient consent and ethical approval granted by the NE London & The City Ethics Committee. Cultures of normal oral keratinocytes (NOK) were grown as previously described [21]. Tumour tissue was minced into pieces of approximately 1 mm^3 ^and incubated for 20 min at 37°C in PBS containing 2.5 mg/ml collagenase I, 0.25% trypsin, and 0.01 mg/ml DNase (all from Sigma, Dorset, UK). Cells for FACS analysis were used either alive or after fixation in ice-cold 70% ethanol.

### Cell culture

Cell lines derived from HNSCC (CA1, H357, 5PT, UK1, CaLH3), breast (MCF7) and prostate (DU145) carcinomas were grown as previously described [19,21]. Cells were plated at clonal density (2000 cells/ml) and individual colonies arising from single cells examined after 5-10 days for differences in morphology and staining for CD44. For comparison of rates of proliferation, H357 cells plated at clonal density were labelled in the S phase of the cell cycle by addition of 5-bromo-2-deoxyuridine (BrdU, Sigma) to culture media to a final concentration of 50 μg/ml for 2 hours. Colonies developed from plated cells were then fixed in 70% ethanol, and stained with an antibody recognizing BrdU adducts (BD Biosciences, Oxford, UK, Cat# 347583) as previously described [19]. For assays of ability to grow as "tumour spheres" in suspension, CA1, CaLH3, H357 and MCF7 cells were grown at clonal density for 4-5 days, removed from the dish by trypsinisation, and sorted to obtain populations of CD44^high ^and CD44^low ^cells. For MCF7 and H357 cell lines, the sorted CD44^high^and CD44^low ^cell subpopulations were then resuspended in conditioned medium with 0.4% low melting point agarose (Sigma) to a cell density of 2000 cells per ml. Cells were plated in triplicate 6 well plates pre-coated with a layer of 1% agarose and left to grow for 2-3 weeks. To quantify sphere formation, 10 random fields of view were taken for each well and the average number of spheres per field calculated. For CA1 and CaLH3 cell lines, the sorted CD44^high ^and CD44^low ^cell subpopulations were plated as single cells in non adherent 48-well plates and left to grow for 3 weeks to grow for sphere formation. Three plates were quantified for each cell line and each cell sub-population, and the data is presented as % of the wells that contained spheres.

### Induction and inhibition of apoptosis

CA1, H357, 5PT, UK1, CaLH3, MCF7, DU145 and NOK cells, were plated at clonal density and after 7-10 days apoptosis was induced by exposure to doses of UVB from 1 to 40 mj/cm^2^, to Cisplatin (50-1000 μM), to neocarzinostatin (NCZ) (10-100 ng/ml), to tumour necrosis factor (TNF, 10 ng/ml), or to etoposide (10-750 μM) (all from Sigma) for 24 or 48 h. Unsorted cells of the CA1, H357 and 5PT cell lines growing *in situ *at clonal density were examined by phase contrast microscopy and were also stained for cleaved Caspase 3 and Annexin V as markers of apoptosis (see below). To assess effects of Chk1 and Chk2 inhibition, debromohymenialdisine (DBH, Axxora, Nottingham, UK) was added to the growth media of the unsorted cells of the Ca1, 5PT, CaLH2 and CaLH3 cell lines grown at clonal density to a final concentration of 8 micromol/ml and cells analysed 18 hours later. Chk1 and Chk2 were also individually down-regulated by transfection with either Chk1, Chk2 or non-targeting siRNAs (ON-TARGETplus SMART pool siRNA, Dharmacon, Inc. Chicago, IL) in unsorted cells of the CA1 and 5PT cell lines with cells stained and assayed 48 h after transfection.

### Recovery after apoptosis-inducing treatment

Sorted cells of the CA1 cell line (CD44^high ^and CD44^low^subpopulations) were plated at the same density, exposed to UV (10 mj) and NCZ (20 ng/ml), left 4 days to recover and then the number of floating (dead) cells was counted. In another experiment, sorted cells of the CA1 cell line (CD44^high ^and CD44^low ^subpopulations) were plated at similar density, exposed to apoptosis-inducing drugs for 48 hours, and then trypsinized and plated again at clonal density. The number of colonies formed was counted in 5 different microscopic fields.

### Immunocytochemistry

Unsorted cultured cells of the CA1, H357, 5PT, CaLH2, CaLH3, MCF7, and DU145 cell lines were fixed in 4% formalin and immunostained as previously described [21]. Primary antibodies were anti-CD44 (1:100, BD Biosciences, Oxford, UK) and anti-cleaved-Caspase-3 (1:250, Promega, Southampton, UK). For Annexin V staining, unsorted cells of the Ca1, H357 and NOK cell lines were grown on culture dishes, washed with Annexin V binding buffer (BD Biosciences) with 1% FBS, incubated for 10 min at 37°C with 10 μl FITC-Annexin V (BD Biosciences) per mL in the same buffer, washed, fixed in 4% paraformaldehide, and mounted in VectaShield-DAPI (Vector laboratories Inc, Burlingame, CA).

### FACS analysis

For assessment of apoptotic sensitivities of CD44^low ^and CD44^high^cell fractions, cells isolated from fresh HNSCC samples were quadruple stained with anti-epithelial-specific-antigen antibody (anti-ESA-APC), anti-CD44 antibody (anti-CD44-PE), AnnexinV-FITC, and DAPI. This allowed distinction of tumour cells from stromal cells, identification of CD44^high ^and CD44^low ^cells, and their further classification into early (AnnexinV^+ ^DAPI^-^) and late (AnnexinV^+ ^DAPI^+^) apoptotic fractions. IgG2ak-PE was used as isotype control. For cell cycle analysis, the cells isolated from fresh HNSCC were fixed and triple stained with anti-ESA-APC antibody, anti-CD44-PE antibody, and PI. Typically, the 3-5% of cells with the highest CD44 expression was designated as CD44^high ^cell subset and the remainder of the population was designated as CD44^low ^cell subset. Cell populations from CA1, H357, 5PT, UK1, CaLH3, MCF7, DU145 and NOK cell lines were triple stained with anti-CD44-PE, AnnexinV-FITC, and DAPI for assessment of apoptosis, or double stained with anti-CD44-FITC and PI for cell cycle assays. To assess apoptosis by loss of mitochondrial membrane potential, a DilC_1_(5) assay (MitoProbe™ Invitrogen, Scotland) was used together with CD44 in a double staining procedure according to manufacturers instructions for CA1, H357 and MCF7 cell lines. To confirm the presence of a G2 block, CaLH3 and H357 cells exposed to various apoptotic-inducing agents were also stained with DAPI, anti-CD44 antibody and anti-Cyclin B1 antibody (H-433, Sc-752, Santa Cruz, Wiltshire, UK).

### IdU pulse chase

To assess cell progression from G2 into G1, unsorted cells of the CaLH3 and H357 cell lines were pulsed with 10 μM 2-Deoxy-5-Iodouridine (IdU) for 20 mins before washing 3 times in PBS and adding fresh medium. Immediately and at 3, 5, 7, and 9 hours, cells were detaching from the flask with Accutase, washed and pelleted, and fixed in ice cold 70% ethanol. Cells were then co-stained with anti-CD44-PE and FITC-conjugated antibody recognizing IdU adducts (BD Pharmingen, Cat# 347583), and processed for FACS analysis. Unless otherwise stated, all FACS reagents were from BD Biosciences (Oxford, UK) and cells were examined using Becton Dickenson LSRII equipment and analysed with FACS Diva software.

### Western blotting

Sorted CD44^high ^and CD44^low ^cells of the CA1 and H357 cell lines, or rapid adherent and late adhering cells of the CA1 and CaLH3 cell lines [15] were isolated after treatment with apoptosis-inducing agents and lysed using RIPA Buffer (50 mM Tris pH 7.3, NaCl, 0.1% SDS, 1%NP40) containing 1% protease inhibitor (Roche Diagnostics, Lewes, UK) and 1% phosphatase inhibitor (Sigma). Protein content was determined using Bio-Rad protein assay (Bio-Rad Laboratories, Hercules, CA, USA). Ten μg total cell protein was mixed with SDS-PAGE buffer and resolved using 4-12% NuPAGE b/Bis Tris Gels (Invitrogen, Renfrew, UK). Proteins were transferred to polyvinylfluoride (PVDF) membranes (Hybond-C Extra, Amersham Pharmacia Biotech, Little Chalfont, UK). Primary antibodies were diluted 1:500 for anti-Chk1 pS317 (Bethyl Lab, TX, USA), 1 μg/ml anti-Chk2 pT26 (Abcam, Cambridge, UK), 1:1000 anti-β-actin (Abcam), and 1:10,000 anti-GAPDH (Abcam).

### Laser capture, RNA isolation and Q-PCR

Colonies of unsorted cells with holo- or para-clone morphologies were laser micro-dissected from the CA1, UK1, and 5PT cell lines, and CD44^high ^and CD44^low ^expressing cells were FACS sorted from primary cultures of human normal oral keratinocytes (NOK). Total RNA was extracted using CellsDirect One-Step qRT-PCR kit, and cDNA generated using SuperScript III first-strand synthesis supermix for qRT-PCR (both from Invitrogen). Q-PCR used a Brilliant SYBR Green kit (Stratagene, La Jolla, CA, USA) and MX3005P apparatus with between 50 and 100 ng of cDNA probed using the following primers: Wee1 - forward: gct ctg tta aac tcc ggg gta, reverse: gac act gtc ctg agg aat gaa g; Myt1 - forward: caa ccg agg ctg tcg aga aa, reverse: ctg tcc aca cca tag ccc t; GAPDH forward: gtg aac cat gag aag tat gac aac, reverse: cat gag tcc ttc cac gat acc.

### Statistical analysis

Numerical data are shown as mean ± SD and are based on a minimum of 3 repeats for each independent biological sample (fresh tissue or cell line). Data was analysed using SPSS version 15 statistical program (SPSS Inc. Chicago, IL, USA), Students t-tests, and analysis of variance (general linear model) with post-hoc tests (Bonferroni multiple comparisons).

## Results

### CD44 expression correlates with *in vitro *colony morphology, clonogenicity and predicts the ability to grow in suspension as "tumour spheres"

**Figure 1:**
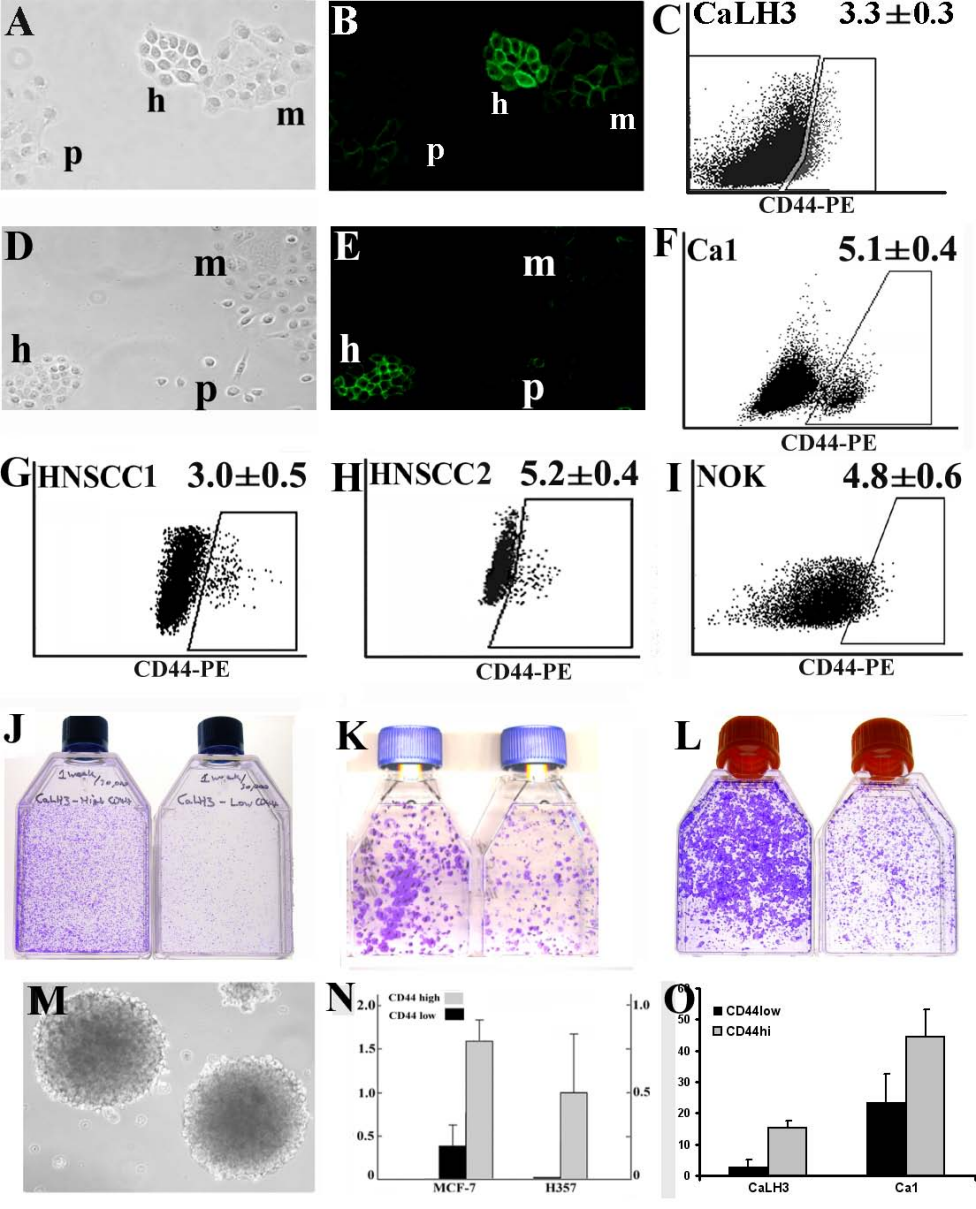
**Identification of subsets of cells with stem-like properties in cells derived from normal and malignant human epithelia**. Holoclone (h), meroclone (m) and paraclone (p) colonies formed by CaLH3 (A) and Ca1 (D) cell lines viewed by phase contrast microscopy and after staining for CD44 (B and E) illustrate the small, refractile, tightly-packed holoclone cells and their strong cell surface staining for CD44. Representative FACS plots for CaLH3 and CA1 cell lines (C and F), two specimens of HNSCC (G, H), and normal oral keratinocytes (NOK) (I). For HNSCC and Ca1 the subpopulation of CD44^high ^formed quite distinct populations, but CaLH3 and NOK showed a more continuous distribution and in this case the 3-5% of cells with the highest CD44 expression was arbitrarily selected as being the subpopulation of CD44^high ^cells. Representative images of cells plated after FACS sorting for CD44 expression in CaLH3 and Ca1 cell lines (J and K) and early (p2) NOK cultures (L). The subpopulation of CD44^high ^cells (left flask in each panel) showed greater clonogenicity than the subpopulation of CD44^low ^cells in both normal and neoplastic-derived cells. Cell lines formed tumour spheres when growing in soft agar (M - representative image of MCF7 cell line), but the CD44^high ^cell fractions formed a significantly larger proportion of spheres than the CD44^low ^fraction when seeded at clonal density in soft agar (N) or when plated as single cells in culture medium in non-adherent plates (O).

Each of the seven human carcinoma cell lines investigated in this study (CA1, H357, 5PT, CaLH2, CaLH3, MCF7, and DU145) developed colonies with morphologies that corresponded, as previously described, to holoclone, meroclone and paraclone colonies (Fig. 1A, 1D). Holoclone colonies showed strong expression of CD44 (Fig. 1B, 1E). When examined by FACS after removal from culture dishes, the cell populations showed various gradients of cell staining for CD44, with distinct sub-populations of CD44^high ^cells found in some cell lines (Fig. 1F). A similar pattern could be observed when analysing carcinoma cells isolated from fresh biopsies of HNSCC (Fig. 1G and 1H). Normal oral keratinocytes showed differential levels of staining for CD44, although they did not show discrete sub-populations of CD44^high ^cells as seen for some malignant cells (Fig. 1I). Sorting cells for high or low levels of expression of CD44 showed, as previously indicated for carcinoma-derived cells [21], that the CD44^high ^cell fractions are more clonogenic when plated in standard culture conditions and form a greater proportion of colonies with holoclone morphologies for both malignant and normal keratinocytes (Fig. 1J, 1K and1L, respectively). Plating the CD44^high ^and CD44^low ^cancer cell fractions as single cells in suspension indicated that the CD44^high ^subpopulation of cells forms a markedly greater proportion of tumour spheres (Fig. 1N and 1O), a property related to tumour initiating ability [22,30].

### Both holoclones and CD44^high ^cell fractions of malignant cell lines are resistant to UVB- and drug-induced apoptosis

**Figure 2:**
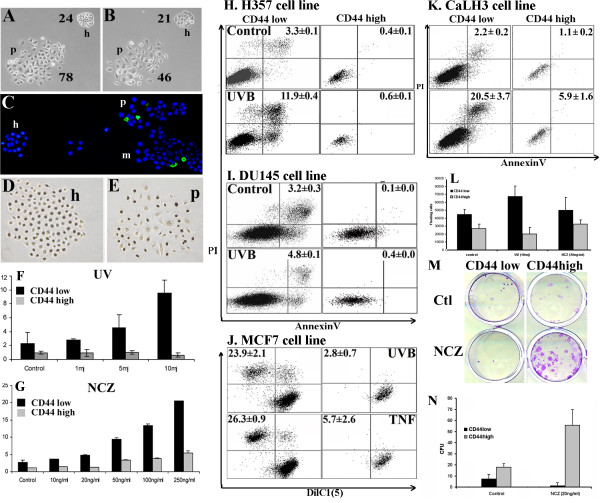
**Analysis of cell death in carcinoma-derived cell lines**. Representative image of holoclone and paraclone colonies of 5PT cell line before (A) and 24 hours after (B) UVB irradiation showing greater loss of cells from the paraclone colony (59.7 ± 8.7% cells remaining, compared to 89.3 ± 10.7% cells remaining in holoclones, p < 0.01). Annexin V of H357 colonies showed higher numbers of apoptotic cells in paraclones (C), while BrdU staining showed similar proliferation rates in both holoclones and paraclones (D, E). Quantification of Annexin V in Ca1 cell line exposed to UVB (F) and NCZ (G) showed dose-related increases of apoptosis for CD44^low ^cells, but markedly lower responses for CD44^high ^cell populations. Representative FACS plots (H, I), showing significantly lower levels of apoptosis among CD44^high ^cells than in CD44^low ^cells (p < 0.01). Apoptosis in the MCF7 breast cell line before and after exposure to 10mj UVB and 10 ng/ml TNF assayed by staining for DilC1(5) and PI (J), showing significantly lower apoptosis in CD44^high ^cells (p < 0.01). Exposure of CaLH3 cell line to very high doses of NCZ (250 ng/ml) showed similar pattern of response as obtained at lower doses of drugs (K). Fewer CD44^high ^cells of Ca1 cell line were found dead (floating) 4 days after inducing of apoptosis when compared with CD44^low ^cells, indicating their increased resistance long term after treatment (L). The recovery of cells after exposure to apoptosis-inducing drugs was significantly higher in CD44^high ^cells compared to CD44^low ^cells of the Ca1 cell line (M, N).

Following UVB irradiation, counts of cells within individual colonies of unsorted HNSCC cell lines indicated significantly greater cell loss from paraclone colonies than from holoclones (Fig. 2A and 2B) and higher number of cells positive for Annexin V (Fig. 2C) and cleaved-Caspase 3 in paraclones and meroclones than in holoclones. Labelling with BrdU confirmed previous observations [19,31] of high levels of cell proliferation in both holoclone and paraclone colonies (Fig. 2D and 2E), indicating that increased survival of cells in holoclones was not simply due to proliferative quiescence. Quantitative assessment of apoptosis by triple-staining with anti-CD44-PE, Annexin V-FITC, and DAPI and FACS analysis showed detectable (baseline) levels of apoptosis in control (un-stimulated) cultures that significantly increased on exposure to UVB, Cisplatin, NCZ, TNF or Etoposide (Fig. 2H, 2I and Table 1). In each of the HNSCC, breast and prostate carcinoma cell lines, the CD44^high ^cell fraction showed significantly lower rates of apoptosis as compared to the CD44^low ^subset (Table 1). Stimulation for various time lengths and concentrations of UVB, Cisplatin, NCZ, or Etoposide consistently induced a time- and dose- dependent apoptosis in the general population, but the rates of apoptosis in CD44^high ^populations remained proportionately lower (Fig. 2F, 2G and Table 1). The proportion of CD44^high ^cells remaining after apoptotic challenge was typically increased (Table 1), indicating the selective survival of this fraction. More significant levels of cell death could be observed in the CD44^high ^fraction of cells when the CaLH3 cells were exposed to very high doses apoptosis-inducing drugs. However, the cell death in this fraction was still at a couple of folds lower levels than in the CD44^low ^fraction of cells (Fig. 2K), displaying the same pattern of resistance to apoptosis-inducing drugs even at these very high doses. DilC_1_(5) release due to the loss of the mitochondrial membrane potential in cells undergoing apoptosis was used as an alternative method to assess cell death, and it also indicated a clear pattern of resistance for the CD44^high ^cell fractions to apoptosis-inducing stimuli (Fig. 2J). Significantly higher numbers of floating (dead) cells were found 4 days after apoptosis-inducing treatment in the pre-sorted CD44^low ^cells than in the pre-sorted CD44^high ^cells of the CA1 cell line, indicating the increased resistance of the CD44^high ^fraction of cells long term after treatment as well (Fig. 2L). Sorted cells of the CA1 cell line (CD44^high ^and CD44^low ^fractions) were exposed to drugs for 48 hours, then trypsinized and plated again at clonal density. CD44^high ^fraction of the cells exposed to NCZ gave rise to significantly higher number of colonies than the CD44^low ^fraction (Fig. 2M, 2N), although for the exposure to UV this was not significantly different.

**Table 1:** Apoptotic and cell cycle data for human carcinoma cell lines.

*Cell line*	*Treatment*	*%CD44^high^*	*% Annexin positive cells in CD44^low^*	*% Annexin positive cells in CD44^high^*	*% cells in G2 in CD44^low^*	*% cells in G2 in CD44^high^*
***Ca1*** ***(n = 3)***						
	
	***Ctrl 24 h***	3.0 ± 0.2	1.4 ± 0.4	0.9 ± 0.1	28.7 ± 4.2	49.1 ± 4.9^##^
	
	***5mJ UV 24 h***	4.0 ± 0.2	3.3 ± 0.1*	1.1 ± 0.1^#^	22.5 ± 0.9	56.0 ± 4.0^##^
	
	***Ctrl 48 h***	3.7 ± 0.6	2.3 ± 1.0	1.0 ± 0.4^#^	23.6 ± 3.6	30.8 ± 1.5^#^
	
	***250 μM CP 48 h***	3.2 ± 0.2	5.5 ± 4.8	2.2 ± 0.5* ^#^	54.2 ± 2.2**	68.9 ± 0.4** ^#^
	
	***20 ng/ml NCZ 48 h***	9.1 ± 0.4*	4.9 ± 0.7*	1.6 ± 0.2^#^	27.2 ± 7.1	49.5 ± 2.8*

***CaLH3*** ***(n = 3)***						
	
	***Ctrl 24 h***	3.1 ± 0.3	2.8 ± 1.2	1.3 ± 0.5^#^	36.4 ± 2.0	42.3 ± 4.1^#^
	
	***5mJ UV 24 h***	3.1 ± 0.3	5.4 ± 0.9*	1.1 ± 0.2^#^	43.1 ± 2.4*	51.7 ± 0.4* ^#^
	
	***Ctrl 48 h***	3.3 ± 0.6	2.7 ± 1.4	1.9 ± 0.6^#^	24.4 ± 9.4	34.3 ± 5.1^#^
	
	***250 μM CP 48 h***	4.1 ± 0.7	22.2 ± 7.8*	19.0 ± 1.8**	66.6 ± 2.8**	73.0 ± 4.1** ^#^
	
	***20 ng/ml NCZ 48 h***	6.2 ± 0.2**	8.8 ± 1.6**	2.3 ± 0.5^#^	18.1 ± 0.7	40.2 ± 2.4* ^#^

***5PT*** ***(n = 3)***						
	
	***Ctrl 24 h***	3.3 ± 0.1	1.3 ± 0.2	1.7 ± 0.4	25.6 ± 0.7	60.7 ± 5.7^##^
	
	***5mJ UV 24 h***	4.4 ± 0.3**	5.5 ± 2.7*	2.6 ± 1.0	37.3 ± 17.0	65.9 ± 10.0^#^
	
	***Ctrl 48 h***	3.1 ± 1.5	1.6 ± 0.5	1.0 ± 0.4^#^	18.7 ± 4.2	31.1 ± 1.9^##^
	
	***250 μM CP 48 h***	3.6 ± 0.9	6.3 ± 0.9**	1.8 ± 0.8^#^	46.0 ± 12.0	66.1 ± 2.6**
	
	***20 ng/ml NCZ 48 h***	3.4 ± 1.1	3.5 ± 0.3**	2.1 ± 0.6* ^#^	17.1 ± 1.1	27.7 ± 2.3^##^

***H357*** ***(n = 3)***						
	
	***Ctrl 24 h***	4.2 ± 1.1	2.5 ± 0.9	1.4 ± 0.4^#^	23.6 ± 5.9	23.3 ± 3.3
	
	***5mJ UV 24 h***	3.6 ± 0.5	11.1 ± 11.6	3.6 ± 2.4^#^	18.3 ± 4.6	33.8 ± 15.0
	
	***Ctrl 48 h***	3.3 ± 1.0	1.3 ± 0.7	1.1 ± 0.7	19.6 ± 4.5	26.9 ± 3.2^#^
	
	***250 μM CP 48 h***	3.9 ± 0.3	8.7 ± 2.7**	2.1 ± 0.5* ^#^	49.7 ± 1.6**	42.4 ± 1.6**
	
	***20 ng/ml NCZ 48 h***	7.9 ± 2.9	5.1 ± 0.8**	2.2 ± 0.8^#^	15.0 ± 1.0	31.5 ± 2.00^#^

**Table key**: Data are presented as mean ± SD of 3 independent assays. * p value < 0.05 when compared with the control group; ** p value < 0.01 when compared with the control group; # p value < 0.05 when compared with the CD44 low population from the same group; ## p value < 0.01 when compared with the CD44 low population from the same group.

### Apoptotic resistance of CD44^high ^cells is associated with longer time spent in the G2 phase of cell cycle

**Figure 3:**
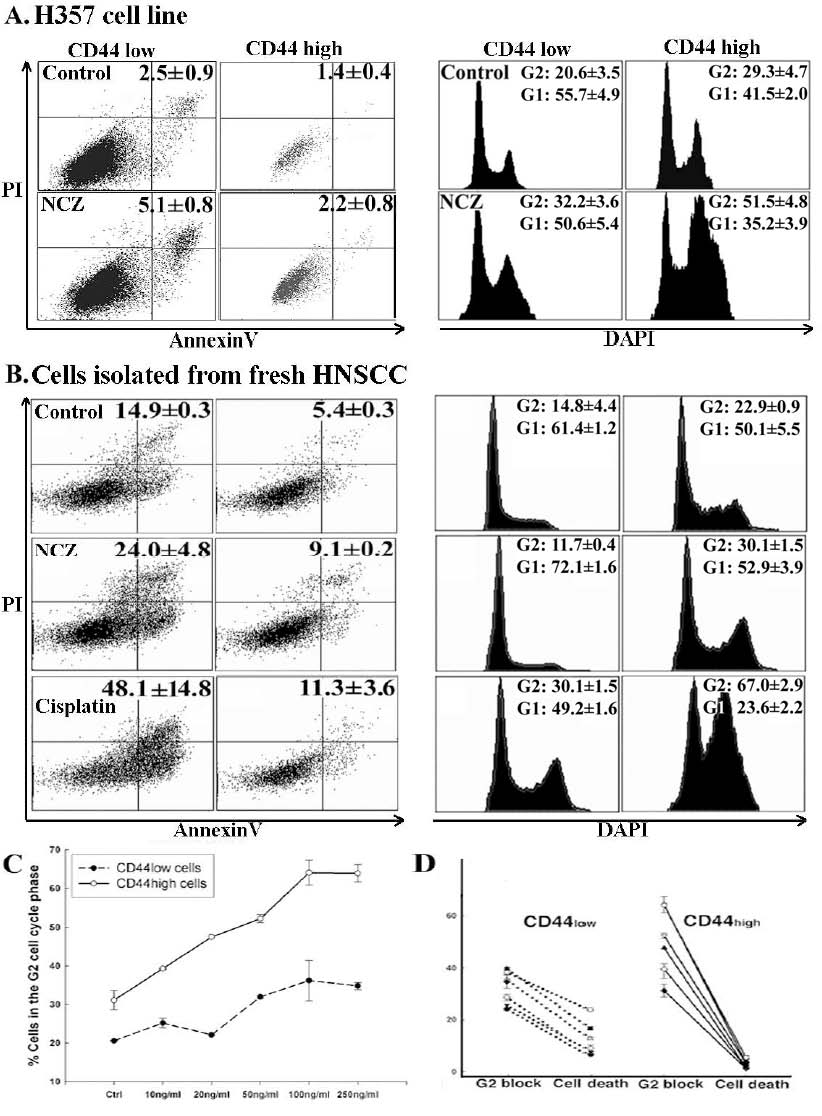
**Apoptosis and cell cycle parameters for CD44^low ^and CD44^high ^cell populations in carcinoma-derived cell lines and fresh biopsies from HNSCC**. Representative FACS plots analysing apoptosis (left panel) and cell cycle (right panel) in CD44^low ^and CD44^high ^sub-populations of H357 cell line exposed to NCZ (A), showing that, in both control cells and cells exposed to 20 ng/ml NCZ, markedly lower levels of apoptosis and greater proportion of cells in the G2 phase of the cell cycle are found among CD44^high ^cells than in the CD44^low ^cells (for both p < 0.01). Data from cells isolated from fresh tumours (B) either untreated, treated with 20 ng/ml NCZ for 24 hours, or 250 μM Cisplatin for 48 hours showed a consistently higher level of apoptosis for CD44^low ^cells than for CD44^high ^cells and consistently larger fractions of CD44^high ^cells in the G2 phase of the cell cycle and smaller fractions in G1 phase of the cell cycle (for both p < 0.05). After treatment with NCZ or Cisplatin the proportion of apoptotic cells in both cell fractions increased but remained 2-4 fold lower for CD44^high ^cells which also showed a marked increase in the proportion of cells in G2. Graph showing the consistent difference in G2 block between CD44^high ^and CD44^low ^sub-populations of Ca1 cell line treated with increasing concentrations of NCZ (C) and the inverse correlation between the proportion of cells in G2 block and the proportion of Annexin V positive cells in CD44^high ^cells at various levels of NCZ exposure (D).

**Figure 4:**
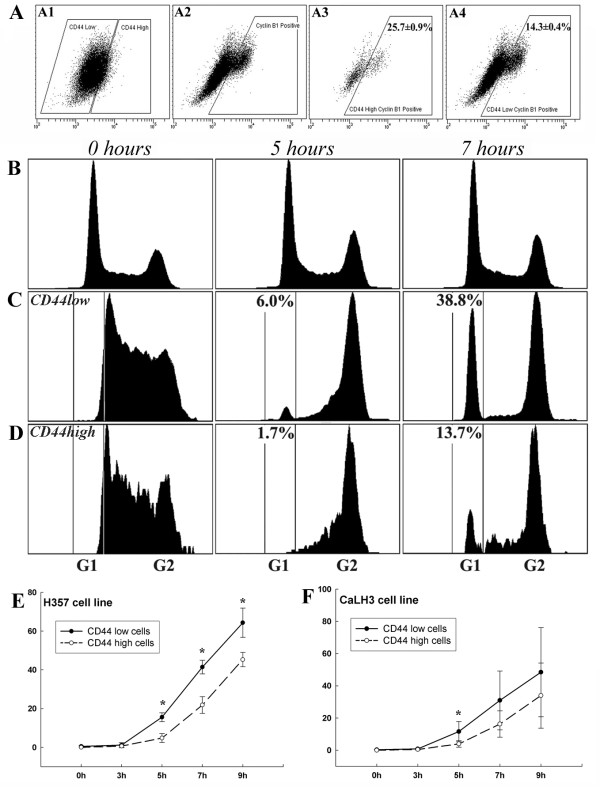
**Cyclin B staining and analysis of passage through G2 by IdU pulse-chase**. FACS analysis of co-staining for CD44 and cyclin B1 of CaLH3 cell line (A): panels A1 and A2 show gates positioned to identify CD44^high^and cyclin B1 positive cells respectively; panels A3 and A4 show the distribution of cyclin B1 positive cells in CD44^high ^and CD44^low ^cell fractions respectively (p < 0.01). The same assay for the Ca1 cell line indicated 49.0 ± 3.5% of CD44^high ^cells expressing cyclin B1 compared to 14.9 ± 0.2% of CD44^low ^cells (p < 0.01). FACS plots of H357 cell line triple stained for DNA content with DAPI and with antibodies against CD44 and IdU adducts showing cell cycle distribution of IdU labelled cells in CD44^high ^and CD44^low ^cell fractions with time after labelling (B, C, D). Typical cell cycle distribution of total cell population at selected time points (B). Cell cycle distribution of CD44^low ^cells exposed to IdU for 60 min prior to fixation (0 hours) showed cells distributed in S and early G2. By 5 hours after labelling, cells began to return to G1, and by 7 hours nearly 40% of cells have re-entered G1 (C). Distribution of IdU-labelled CD44^high ^cells after initial labelling (0 hours) showed cells distributed in S and G2 as for CD44^low ^cells. By 5 hours after labelling fewer cells have returned to G1 and by 7 hours the number was still lower (D). Data for all time points are summarised for H357 (E) and CaLH3 (F) cell lines.

FACS analysis after staining the cell populations for CD44 and DNA content indicated significant cell cycle differences between CD44^high ^and CD44^low ^cells (Fig. 3A and Table 1). In un-stimulated cells, the proportion of cells in the G2 phase of the cell cycle was consistently and significantly higher for the CD44^high ^subset of cells than for the CD44^low ^cells (Table 1), at the expense of G1 phase of the cell cycle (Fig. 3). Treatment with NCZ or Cisplatin induced small increases in the fraction of CD44^low ^cells in G2 but significant increases in the proportion of CD44^high ^cells (Fig. 3C). Exposure to increased concentrations of NCZ showed that the rates of apoptosis correlated inversely with the extent of the G2 block in the CD44^high ^cells (Fig. 3D). For the H357 and CaLH3 cell lines the proportion of cells present in the G2 phase was also assessed by an alternative method of quantifying the expression of Cyclin B1, a molecule predominantly expressed in G2/M phase. These cell lines showed a significantly greater (2-3 fold greater, p < 0.01) number of cell expressing Cyclin B1 in the CD44^high ^cell fractions (Fig. 4A).

### CD44^high ^cells freshly isolated from HNSCC are resistant to drug-induced-apoptosis and spend longer in the G2 phase of the cell cycle

Cells freshly isolated from specimens of HNSCC were examined immediately and at 24 and 48 h after treatment with Cisplatin or NCZ. Negative staining for ESA was used to exclude non-epithelial cells (*i.e*., fibroblasts, inflammatory cells, *etc*.) from the analysis. All samples showed a range of CD44 staining levels and some specimens showed distinct sub-populations of 3-5% of cells with high CD44 staining (Fig. 1D and 1E). The rates of apoptosis were relatively higher for the un-treated carcinoma cells isolated from fresh tumour biopsies then for un-treated cultured carcinoma cell lines, but when the CD44^high ^and CD44^low ^cell fractions were separately analysed, the rates of apoptosis were found to be consistently lower for the CD44^high ^cell fractions than for the CD44^low ^fractions (Fig. 3B, Table 2). This difference was statistically significant (p < 0.05) for 6 of the 8 individual tumour specimens examined for apoptosis. FACS analysis after staining cell populations for CD44 and DNA content indicated significant cell cycle differences between CD44^high ^and CD44^low ^un-treated cells in all tumours (Fig. 3B, Table 2), with up to a 2-fold greater number of CD44^high ^cells in the G2 phase of the cell cycle and lower cell numbers in the G1 phase of the cell cycle. Treatment with NCZ or Cisplatin (Fig. 3B) consistently increased overall apoptotic rates, but the apoptotic rates for CD44^high ^cells invariably remained a couple of folds lower than for the rest of the population. After apoptotic challenge an increased fraction of CD44^low ^cells was also found in G2, but consistently greater increases were found for CD44^high ^cells. Following apoptotic stimulation by exposure to Cisplatin and NCZ, the size of the remaining CD44^high ^cell fractions typically increased, suggesting again selective survival of the CD44^high ^cells (Table 2).

**Table 2:** Apoptotic and cell cycle data for cells derived from head and neck human oral carcinomas and from normal human oral mucosa.

*Cell type*	*Treatment*	*%CD44^high^*	*% Annexin positive cells in CD44^low^*	*% Annexin positive cells in CD44^high^*	*% cells in G2 in CD44^low^*	*% cells in G2 in CD44^high^*
***HNSCC derived cells*** ***(n = 11)***						
	
	***Ctrl 24 h***	3.9 ± 0.4	15.1 ± 11.5	8.9 ± 8.9	12.9 ± 1.2	23.0 ± 2.2^##^
	
	***250 μM CP 24 h***	6.8 ± 1.0*	38.5 ± 19.5	22.1 ± 16.7	15.0 ± 2.1	41.1 ± 9.7^#^
	
	***20 ng/ml NCZ 24 h***	6.0 ± 0.8	19.9 ± 10.3	3.5 ± 2.8	11.6 ± 1.9	31.4 ± 6.0^#^
	
	***Ctrl 48 h***	4.3 ± 0.7	14.9 ± 7.6	2.5 ± 1.6	14.1 ± 3.1	23.5 ± 5.3
	
	***20 ng/ml NCZ48 h***	11.0 ± 3.9**	36.3 ± 16.3	11.0 ± 4.8	17.1 ± 2.9	76.9 ± 23.2**

***NOK*** ***(n = 5)***						
	
	***Ctrl 24 h***	4.8 ± 1.7	5.2 ± 0.9	0.7 ± 0.4^##^	10.4 ± 1.9	20.9 ± 2.6^#^
	
	***10mJ UV 24 h***	7.5 ± 2.8	7.5 ± 2.8	1.3 ± 0.5^#^	28.9 ± 2.1*	44.9 ± 4.7^##^
	
	***Ctrl 48 h***	3.3 ± 0.6	15 ± 4.5	2.1 ± 0.3^#^	11.6 ± 1.8	16.0 ± 2.3
	
	***250 μM CP 48 h***	4.6 ± 0.9	28.8 ± 4.0	3.6 ± 0.5* ^##^	15.8 ± 4.0	28.6 ± 2.3

**Table key: **Data are presented as mean ± SD of 3 independent assays. * p value < 0.05 when compared with the control group; ** p value < 0.01 when compared with the control group; # p value < 0.05 when compared with the CD44 low population from the same group; ## p value < 0.01 when compared with the CD44 low population from the same group.

### CD44^high ^cells transit the G2 compartment more slowly

The greater proportion of CD44^high ^cells observed to be present in G2 could result from a general increase in the time taken for the CD44^high ^cells to transit G2 or from a more permanent block of a sub-fraction of the CD44^high ^cell population. To assess the time taken to transit G2, cultures of the H357 and CaLH3 cell lines were exposed to IdU to label the cells in the S phase and the time taken for these cells to transit through G2 and return to G1 was then determined by sequential FACS analysis of cell cohorts (Fig. 4B, C, D). Five hours after IdU labelling, a higher proportion (15.6 ± 2.3% and 11.6 ± 6.2% for H357 and CaLH3 respectively) of CD44^low ^cells (Fig. 4C) was found to return to G1 as compared to CD44^high ^cells (4.8 ± 2.3% and 3.9 ± 2.0% respectively, p < 0.05) (Fig. 4D), indicating that although both CD44^high ^and CD44^low ^cells transit G2, CD44^high ^cells do so more slowly. Results from four data series for each cell line estimate the return of CD44^high ^cells to G1 to take 1.5 -2.0 hr longer than for CD44^low ^cells even in un-treated control cultures (Fig. 4E and 4F).

### Survival of CD44^high ^cells is impaired by reducing the time spent in G2

**Figure 5:**
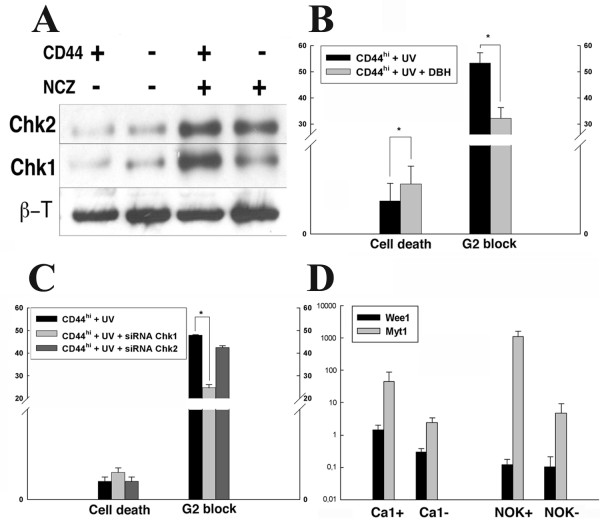
**Analysis of components of the G2 block**. Western blot of CD44^high ^and CD44^low ^fractions showing low baseline levels of phosphorylated Chk1 and Chk2 in the unstimulated Cal cell line (A). After 24 hours of exposure to 100 ng/ml NCZ levels increase, especially for CD44^high ^cells (A). Cells of the Ca1 cell line before and after inhibition of Chk1/2 with DBH show increased cell death associated with partial release from the G2 block (p < 0.01) (B). Rates of cell death and proportion of CD44^high ^cells after independent knockdown with siRNAs of Chk1 and Chk2 in CaLH3 cells show an effect mainly via Chk1 (C). Message levels for Wee1 and Myt1 in CD44^high ^and CD44^low ^cells of the Ca1 cell line. The CD44^high ^cells of both cell types show higher levels of Myt1 and CD44^high ^Ca1 cells higher levels of Wee1 (D).

Comparison of all data sets for CD44^high ^and CD44^low ^cells in variously-treated individual cell lines indicated that lower cell death was associated with larger fractions of CD44^high ^cells present in the G2 phase of the cell cycle (Tabel 1 and Fig. 3D). G2-cell cycle arrest is often associated with phosphorylation of the Chk1 and Chk2 check-point proteins and both the CD44^high ^and CD44^low ^cells, and the rapid and late adhering cells of un-stimulated populations contained low levels of phosphorylated Chk1 and Chk2 (Fig. 5A). After treatment with NCZ, their levels increased but the CD44^high ^and the rapid adhering cell fractions showed a higher increase, suggesting greater checkpoint activation in CD44^high ^or rapid adhering cells in response to DNA damage. Treatment with DBH, a broad inhibitor of Chk1/2 checkpoint actions, resulted a significant decrease in the percentages of CD44^high ^cells in the G2 phase of the cell cycle, and this reduction was associated with significantly (p < 0.05) increased rates of death of CD44^high ^cells after exposure to UV (Fig. 5B). Targeting Chk1 with siRNA significantly (p < 0.05) decreased the fraction of CD44^high ^cells in G2 and increased their rate of cell death but little effect was seen after targeting Chk2 (Fig. 5C). QPCR showed that CD44^high ^cell fractions or cells laser captured from holoclones had higher levels of transcription of Wee1 and Myt1, the up-stream molecules involved in activation of the G2 checkpoint response (Fig. 5D).

### Apoptotic sensitivity of normal oral keratinocytes

**Figure 6:**
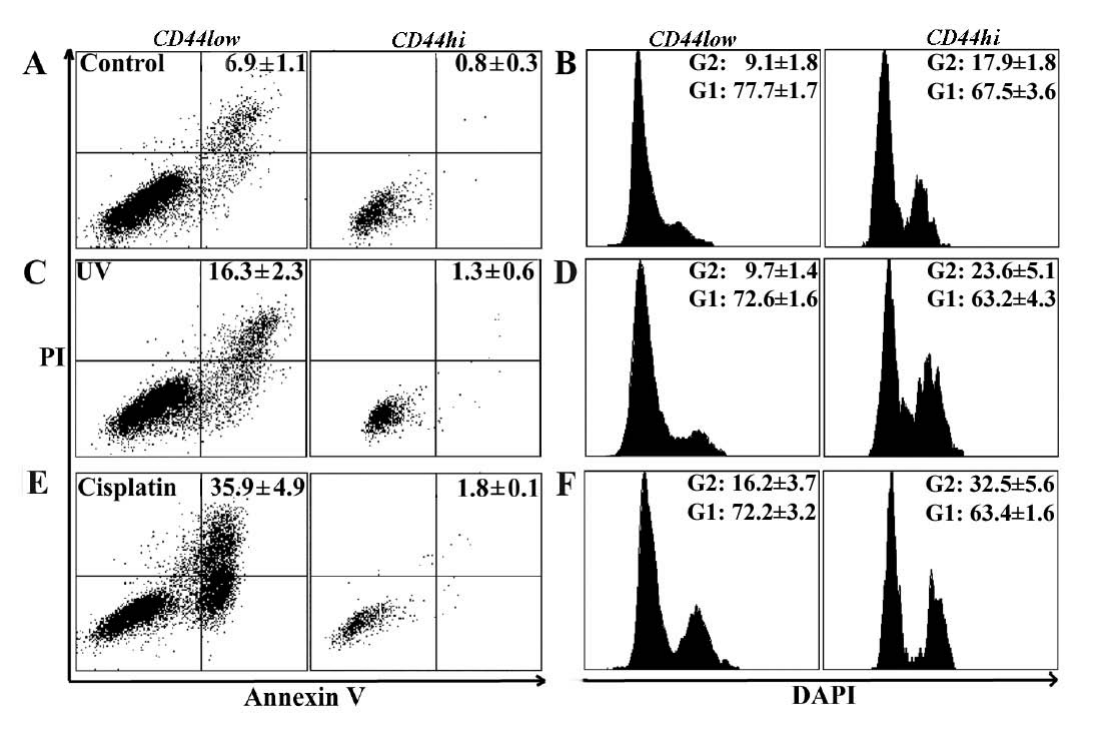
**Apoptosis and cell cycle parameters for sub-populations of NOK**. Apoptotic rates and cell cycle parameters for CD44^high ^and CD44^low ^cell fractions of early passage normal oral keratinocytes. CD44^high ^cells (right panel of each pair) show lower rates of apoptosis and a higher proportion of cells in G2 (both p < 0.05) than the CD44^low ^sub-population (left panel of each pair) (A, B). Treatment with UVB and Cisplatin increases apoptosis (C, E), but the proportion of CD44^high ^cells in apoptosis remains markedly lower (p < 0.01). UVB and NCZ treatment is also associated with increases in the proportion of CD44^high ^cells in G2 and decrease in G1 phase of the cell cycle (D, F).

Murine epithelial stem cells appear highly susceptible to apoptosis [26,27] but there is little information about differential apoptotic sensitivities of stem and amplifying fractions of normal human oral keratinocytes (NOK). To examine whether the greater apoptotic resistance of malignant CD44^high ^cells is related specifically to malignant change, or is a general a property of human epithelial stem cells, low passage cultures (P3-4) of NOK isolated from normal human oral mucosa were examined (n = 5). Analysis of the 3-5% of NOK cells with the highest CD44 expression showed that these cells, as for the CD44^high ^malignant cells, were more resistant than the remainder of the population to apoptotic induction and spent greater time in the G2 phase of the cell cycle (Fig. 6). Baseline levels of apoptosis increased markedly in NOK cultures after exposure to UVB, Cisplatin, NCZ, and Etoposide (Fig. 6 and Table 2) but the proportion of apoptotic cells in CD44^high ^cell fractions consistently remained lower and the time spent in G2 became further extended.

## Discussion

The present study found that CD44^high ^cells isolated from fresh biopsies of HNSCC show greater resistance to drug-induced apoptosis than cells with lower levels of CD44 expression and that this enhanced resistance is associated with longer time spent in the G2 phase of the cell cycle. The presence of such differences between CD44^high ^and CD44^low ^cells in cell populations freshly isolated from carcinomas indicates their prior existence within the *in vivo *tumours of origin. Similar differences between CD44^high ^and CD44^low ^cells, and also between cells from holoclones and paraclones, were also consistently found in all cell lines investigated in the study. Cells freshly isolated from tumours tended to show somewhat higher overall rates of apoptosis than those for cell lines but it is unclear to what extent this reflected intrinsic differences between carcinoma-derived cell lines and cells freshly isolated from carcinomas. It could be due to the adaptation of cell lines to *in vitro *conditions, but it could also result from the more extensive enzymatic dissociation needed for the fresh tumours that may have affected the apoptotic response or even induced some degree of apoptosis in the isolated cells. Nevertheless, taken together, the findings of similar patterns of apoptotic resistance of CD44^high ^cell fractions in fresh HNSCC cells, HNSCC cell lines, and also for cell lines derived from breast and prostate malignancies, suggest that this pattern is likely to be typical of a wide range of carcinomas.

Enhanced susceptibility of normal epithelial stem cells to apoptosis has been proposed to act as a defence against malignancy by eliminating stem cells that have acquired even minor DNA damage [31]. Normal murine epithelial stem cells show differentially high rates of apoptosis both *in vitro *and *in vivo *[31,32] and the development of murine skin cancer has been associated with acquisition of stem cell resistance to apoptosis [33]. We therefore initially anticipated that the enhanced apoptotic resistance found for malignant CD44^high ^cell fractions was likely to be a result of malignant progression causing a shift of relatively low apoptotic resistance of normal stem cells to relatively high resistance of malignant stem cells. The finding of greater apoptotic resistance of CD44^high ^cells in cultures of human NOK, apparently the reverse of that for murine cells, was therefore unexpected. What effects such differences between the apoptotic behaviours of normal human and murine epithelial stem cells may have on patterns of carcinogenesis remain to be determined. However, the apoptotic resistance of CD44^high ^cells in early passage cultures of NOK indicates that enhanced stem cell resistance in human malignancy is not a *de novo *consequence of the malignant change but represents enhancement of a pre-existing epithelial stem cell property.

Various mechanisms of greater resistance of cancer cells with stem-cell properties to apoptosis have been previously suggested. Surprisingly, proliferative quiescence and presence of ABC transporters, two properties that have often been related to therapeutic resistance of cells with stem-like properties, did not appear to be associated with the survival of CD44^high ^cells *in vitro*. Proliferative quiescence was suggested to be radio- and chemo- protective and appeared to protect leukemic progenitor cells from therapeutic actions [34]. Little is known about the *in vivo *proliferative rates of cells with stem-like properties in carcinomas, as compared to the bulk of the tumour, but here we show *in vitro *that the CD44^high ^cells had greater apoptotic resistance despite their high proliferative rates indicated by cell labelling and time-lapse video (data not shown). High expression levels of ABC transporters were also suggested to be chemo-protective [35] and high expression levels were reported for cancer cells with stem-like properties [21,36,37]. Apoptotic challenge with UVB was therefore initially chosen in this study to provide a well-controlled apoptotic stimulus unlikely to be affected by ABC status. Little difference was subsequently detected between the responses of cells to UVB and to several chemical agents suggesting that differences in ABC status may not be important under the *in vitro *conditions employed.

The present study showed a consistent relationship, for all types of cells examined, between lower rates of apoptosis and a higher proportion of cells in G2. In line with previous studies of glioblastomas [24,27], these data indicates that the selective resistance of cells with stem-like properties (CD44^high ^or rapid adhering cells) to apoptosis appears to be mediated through preferential activation of cell cycle checkpoints in response to DNA damage, and that these are reduced after inhibition of Chk1/Chk2 kinases. However, it is likely that several mechanisms co-operate to produce selective apoptotic resistance of carcinoma cells with stem-like properties in cancers. Work on breast cancer cell lines suggests that the radio resistance of the CD44+/CD24- subset of cells with stem-like characteristics might be related to increased activation of Notch [28], a molecule reported to protect normal and breast cancer cells against diverse apoptotic stimuli [38]. High levels of expression of the anti-apoptotic molecule survivin were also reported in CD44+/CD24-/low fractions of breast cancer lines [39] while high expression of survivin was observed in the stem cell fraction of normal keratinocytes [40], suggesting that this may be an anti-apoptotic mechanism common to a range of epithelial stem cells. Interestingly, several molecules shown to be expressed at higher levels on the surface of epithelial cells with stem-like properties, including CD44, EGFR and ESA, are typically up-regulated in epithelial malignancies, are associated with reduced apoptotic activity, and have interactions that augment their individual anti-apoptotic effects [41-43]. Determining what degrees of resistance each mechanism typically confers, and how such mechanisms may differ between individual tumours, requires further work however.

## Conclusions

The findings of this study provide support for the concept that clinical failure to eliminate carcinomas with chemo- and radio-therapy may be related to unique properties of a sub-fraction of the total tumour cells that has stem-like properties. These cells were demonstrated, by various methods, to have a tendency to dwell longer in G2, the phase of the cell cycle associated with DNA repair, and release from G2 can be used to push them into apoptosis. Differential apoptotic resistance of cells with stem-like properties was also found in cultures of normal oral keratinocytes indicating that such resistance is not a *de novo *property acquired with the malignant change but a constitutive feature of stem-like epithelial cells. Its presence in freshly isolated HNSCC also indicates that it is not simply an adaptation to *in vitro *conditions, suggesting that malignant cell lines may provide an efficient and effective preliminary means of studying malignant stem-cell-like properties and may also provide systems for analysing and devising new methods for their elimination.

## Competing interests

The authors declare that they have no competing interests.

## Authors' contributions

LJH carried out the UVB, TNF, Etoposide and Cisplatin apoptosis and cell cycle studies in head and neck and breast carcinoma cell lines and some strains of the normal epithelial cells, Dil5 staining, and the DBH inhibitory experiments. DEC carried out the Cisplatin and NCZ apoptosis and cell cycle studies in cells isolated from fresh tumours, *in situ *Annexin-V staining and the siRNA studies, participated to western blotting, qPCR, and statistical analysis. LG carried out the apoptosis and cell cycle studies in prostate carcinoma cell line and some strains of the normal keratinocytes, the pulse-chase experiments, cleaved Caspase 3 staining, cyclin B1 staining and participated to western blotting, and statistical analysis. BF performed the immunofluorescent staining. AB participated to qPCR. ICM conceived and coordinated the study. All authors participated in drafting the manuscript, read and approved the final manuscript.

## Pre-publication history

The pre-publication history for this paper can be accessed here:

http://www.biomedcentral.com/1471-2407/10/166/prepub
